# Enduring the Unbearable—A Phenomenological Hermeneutical Study on Nurses' Lived Experiences of Stress During the COVID‐19 Pandemic

**DOI:** 10.1111/scs.70062

**Published:** 2025-07-08

**Authors:** Hillewi Carnesten, Petra von Heideken Wågert, Lena Wiklund Gustin, Karin Skoglund, Christina Andreae

**Affiliations:** ^1^ School of Health, Care and Social Welfare Mälardalen University Eskilstuna Sweden; ^2^ Department of Health and Care Sciences UiT/The Arctic University of Norway Narvik Norway; ^3^ Centre for Clinical Research Sörmland Uppsala University Eskilstuna Sweden

**Keywords:** compassion, emergency care, hospital, lifeworld, moral stress, stress of conscience, stressors, work environment

## Abstract

**Background:**

The COVID‐19 pandemic continues to pose significant challenges to the welfare system by stress‐related ill health and increased turnover rates among nurses.

**Aim:**

The aim of this study was to explore nurses' lived experiences of stress in the transformed caring reality during the COVID‐19 pandemic.

**Methodological Design and Justification:**

Caring theory and phenomenological hermeneutical philosophy guided this interpretative study. Findings comprise a broadened understanding of stress experienced by nurses.

**Ethical Issues and Approval:**

Ethical approval was granted from the Swedish Ethical Review Authority. All participants provided informed consent to participate beforehand.

**Research Method:**

In‐depth audio recorded interviews with 13 nurses were performed and transcribed verbatim. Analysis was guided by the three steps of Lindseth and Norberg: naïve understanding, structural thematic analysis and comprehensive understanding.

**Results:**

In this study, one main theme *Enduring the unbearable* emerged, and was illuminated in the major themes: Being deprived of the caring self; Seeing self‐neglect as the way forward and Feeling vulnerable and unsupported. The interpreted meaning of nurses' lived experiences of stress during the COVID‐19 pandemic accommodates the dilemma of enduring the unbearable whilst being subjected to silencing one's inner ethical, caring compass.

**Conclusions:**

The results underscore the substantial and persistent stress related health risks nurses faced during the COVID‐19 pandemic and emphasise the necessity of integrating ethical considerations into policies designed to support nurses' health in future emergencies.

## 1 Introduction

Over the last decades, occupational stress‐related ill health has become a growing problem within healthcare both in Sweden and internationally. From a caring science paradigm, nurses' ultimate responsibility is to bestow holistic care to relieve patients' suffering and restore health. Thus, being a nurse means to inevitably be subjected to stress by encountering tragedy and death. The elusive character of caring has been developed by Eriksson [1] in the caritative caring theory mediating caring as ‘to tend, play and learn in faith, hope and love’ in a dignified act of love. Eriksson's theory emphasises the significance of a holistic approach acknowledging patients' suffering and understanding their needs. Eriksson emphasises the importance of alleviating suffering through caring actions encompassing the physical, emotional and existential dimensions of the patient's experience. Holistic understanding of the patient's needs and the role of caring embracing those needs can relieve suffering. This holistic approach corresponds with Benner and Wrubel's theory, which highlights the significance of caring as fundamental to human recovery and healing [2].

The holistic perspective is, aside from being pivotal aiming to relieve patients' suffering, also crucial for nurses to endure encounters with suffering patients. Theorists enhance the importance of ability and circumstances to care for patients according to one's inner ethical compass [1, 2, 3, 4]. Eriksson [1] illuminates in the caritative theory, the endeavour of finding ‘the why’ in suffering. This can be understood as to find alleviation in maintaining one's caring self and professional identity despite facing overwhelming demands.

## 2 Background

In the beginning of March 2020, the stressful work environment in healthcare was exacerbated by the swift spread of the new coronavirus (SARS‐CoV‐2) subjecting frontline nurses to challenges unparalleled in our time. To meet urgent care needs, hospitals rapidly converted selected departments to designated COVID‐19 wards reassigning nurses from many different specialties [5]. Nurses were required to deliver frontline, highly specialised care that exceeded their usual scope of experience and professional competence, often at short notice and with significant risk to their own health [6]. Early international meta‐studies show that working as a nurse in the pandemic frontline is associated with a high incidence of symptoms of stress‐related ill health caused by the extraordinary burden [7, 8, 9]. Even when the pandemic is over, the considerable mental and physical impact among nurses is still present and adds to pre‐pandemic problems with maintaining a healthy sustainable work life for nurses [7, 10, 11, 12].

Stress among nurses has previously been found strongly connected to workload and a high pace work environment [13]. Thereto, unsupportive relations, avoidance of what is perceived as burdensome, and negative perception of the care provided have a negative impact on nurses' health [14]. Hence, in the early pandemic aftermath, a growing body of knowledge illuminate nurses' dichotomous experiences between exhaustion and meaningfulness [15, 16]. The emerging picture of nurses' experiences during the COVID‐19 pandemic is far from one‐sided, and stress to nurses reaches beyond chores and workload [15].

Although the full picture of nurses' experiences facing the COVID‐19 pandemic is still to be drawn, it is clear that caring for patients during this period subjected nurses to immense work life challenges [17]. Stress and its management walk hand in hand. According to Eriksson's theory, enduring stress can be understood as the restoring of meaning, to remain resilient and preserve self‐efficacy and job‐satisfaction despite facing difficult challenges. Resilience is supported by self‐compassion and organisational factors such as constructive leadership and person‐centred care. This has been shown achievable by different means, yet challenged by increased stress‐related ill health among nurses and problems with turnover, decreased quality of care and jeopardised patient safety [18]. Moreover, self‐compassion is considered essential for enhancing personal development and job satisfaction, as well as alleviating stress in nursing practice [19]. Thereto, organisational factors, as constructive and attentive leadership, together with person‐centred care are associated with reducing stress of conscience enhancing nurses' ability to cope [20]. Research propose mentorship programs providing structured orientation and support, fostering a positive work environment and encouraging self‐care practices as coping strategies [21].

Historically, pandemics tend to arise unexpectedly with unpredictable regularity, leaving a hovering threat of their recurrence. In the early COVID‐19 pandemic aftermath, the World Health Organisation (WHO) emphasises the risk of more pandemics to come and long‐lasting consequences in healthcare. The picture mediated by WHO is clear; turnover rates and nursing shortages due to occupational stress‐related ill health increase worldwide. This calls for our study, taking the stance of a lifeworld perspective when exploring nurses' lived experiences of stress when caring for patients during the pandemic. This study aimed to explore nurses' lived experiences of stress in the transformed caring reality during the COVID‐19 pandemic.

## 3 Research Objectives

### 3.1 Design

This qualitative lifeworld led study employed a phenomenological hermeneutical approach guided by Lindseth and Norberg [22, 23].

### 3.2 Study Participants and Setting

This study was conducted during February 2021 to May 2021 in a Swedish region pervasively affected regarding the impact on healthcare workers' work environment. Eligible for inclusion were registered nurses (RNs) with permanent employment the first wave of the COVID‐19 pandemic, March 2020 to September 2020. A convenient, purposive sample of RNs (*n* = 13) were recruited from emergency care units including newly created medical‐, advanced‐ and intermediate COVID‐19 care units, all treating patients with COVID‐19.

### 3.3 Data Collection

Inspired by an interpretative lifeworld philosophy [22, 23] qualitative semi‐structured, face‐to‐face, in‐depth interviews (*n* = 13) with an interview guide were conducted in secluded rooms at the hospital. To encourage rich descriptions the participant was asked to ‘*describe an event that you remember particularly well from the pandemic*’. Clarifying questions were asked when needed. The audio‐recorded interviews were performed by the first and the last author, lasted from 42 to 75 min and were then transcribed verbatim.

### 3.4 Data Analysis

Guided by Lindseth and Norberg's phenomenological hermeneutical analysis method interpretation followed three phases: naïve reading, structural thematic analysis and comprehensive understanding in an interpreted whole [22, 23]. Initially, the text was read several times to grasp its superficial instant meaning, undertaken by open, reflective reading while taking notes. Two researchers performed a naïve reading where interpretations were separately formulated in naïve understanding of what the texts where about. Then the texts were imported into a qualitative data analysis software program (N'vivo, version 1.6) that helped organise data into folders and groups and keep everything structured and easily accessible. Analysis in N'vivo began by two researchers' independently thoroughly reading each text, encompassing and highlighting meaning units of variated lengths, all with significant information. Meaning units were then clustered into shorter phrases, condensed and assigned appropriate codes relevant to the research questions. Codes are labels that capture the essence of the information in the condensed meaning units.

Next, codes were merged into subthemes and themes, see Table 1. In this process, Word documents were used to display the emerging themes instead of N'vivo. Throughout the process structural explanation and phenomenological interpretation was undertaken in a dialectic movement between parts and the whole in the texts. By reflecting on the subthemes and themes in the entire research group, and by mirroring the naïve understanding a new comprehensive understanding emerged, see Table 2. To enable bridling of preunderstandings this process was undertaken by a decontextualization addressing the text as a world of its own. Findings were then recontextualized and related to theory. The expertise from all the researchers involved was pivotal reaching a new comprehensive understanding.

**TABLE 1:** Results overview.

Sub‐themes	Major themes
Trespassing one's ethos and quietly obey orders	*Being deprived of the caring self*
Violating the core of caring	
Losing control and feeling inadequate	
Continuing to work despite a sore, aching body	*Seeing self‐neglect as the way forward*
Continuing to work by shutting oneself and the outside world out	
Being enclosed in an emotional vacuum	
Being forced into a frightening, stressful and demanding reality	*Feeling vulnerable and unsupported*
Being responsible for advanced care without training	
Feeling left out and enduring challenges alone	

**TABLE 2:** Example of the process from meaning unit to theme.

Meaning unit	Condensation	Code	Sub‐theme	Theme
‘*You were just thrown into it. No one to learn from. That's how it was. Completely crazy*’.	Being subjected to advanced care with no support which felt completely crazy.	Advanced responsibilities and lack of support	Being responsible for advanced care without training	*Feeling vulnerable and unsupported*

## 4 Results

The results are presented following the three steps of the phenomenological hermeneutical method. Interpretations are guided by Eriksson's [1] caritative theory emphasising the indivisible human being, ethical and moral dimensions of caring, and the patient as a suffering human needing care.

### 4.1 Naïve Understanding, Grasping What the Text Is About

Nurses' experiences of the transformed caring reality during the COVID‐19 pandemic can be understood as an unprepared confrontation with an unknown, frightening and demanding world. The transformed reality imposes high demands and extensive responsibilities, resulting in a profound sense of loneliness and self‐doubt among nurses. The depletion of nurses' bodily resources due to exhaustive work and the absence of recovery further exacerbates stress. The pandemic necessitates that nurses endure unpredictable challenges with minimal guidance. Feelings of alienation arise, prompting existential questions such as, ‘Is this the real world?’ Additionally, nurses experience guilt and conflict due to their inability to alleviate patients' suffering, which fosters a longing for improved working conditions and necessary prerequisites to provide holistic care to suffering patients. The reward is witnessing patients recover, which entails a sense of pride in being part of the frontline workforce. However, the remuneration is insufficient relative to the magnitude of their contributions.

### 4.2 Structural Thematic Analysis

This section illuminates the structured analysis and the connections between parts, patterns and themes outlining how relationships relate to each other. Nuanced interpretations are presented, subsequently leading to a comprehensive understanding of the phenomenon under study.

#### 4.2.1 Being Deprived of the Caring Self

The theme being deprived of the caring self includes trespassing one's ethos and quietly obey orders and violating the core of caring and losing control and feeling inadequate.

*Trespassing one's ethos and quietly obey orders* mean caring for patients in unsustainable conditions and compelling oneself to inadequacies and unworthy care. Unattended difficult emotions in conflict with one's ethos are a numb way of living and one is cut off in a borderland filled with demands and uncertainty while having nobody who listens:

Because we did wrong. But I felt like I was just to abide and do what they said. Even though you felt it was wrong, you did it… if they said we can't use protection equipment. Then we didn't use it.

*Violating the core of caring* mean harbouring feelings of guilt and living in conflict as one's ethical compass is scrutinised by patients sometimes being kept alive in an undignified manner. One's caring self as a present and compassionate nurse aiming to relieve suffering is challenged by the forced distance. New assigned work tasks mean increased and changed responsibility lacking prerequisites for holistic caring. Lack of knowledge on how to perform put patients at risk, lack of resources to protect patients and oneself and lack of support altogether deplete nurses' resilience and ability to cope. Being forced to refrain from bestowing patients' basic needs, either unprioritized or impossible to manage, mean subjecting oneself to self‐criticism and frustration:

Some were too ill to be turned in bed…There was a lot of inadequacy in general. On one's own account, but also for the patients.

*Losing control and feeling inadequate* mean harbouring strong, irrevocable memories of suffering patients and relatives adding to feelings of guilt. Work determined by others lacking instructions on how to care for patients and lack of protective equipment add to the ethical struggle to alleviate suffering. Thereto, being assigned work tasks one lacks competence for is a feeling of free falling without support:

You had all these new technical devices and drains and all that, you felt like….“what if something happens and then I'm standing there, and maybe it's only a matter of minutes…and I don't know what to do?”

#### 4.2.2 Seeing Self‐Neglect as the Way Forward

Seeing self‐neglect as the way forward includes continuing to work despite a sore, aching body; continuing to work by shutting oneself and the outside world out and being enclosed in an emotional vacuum.

*Continuing to work despite a sore, aching body* means one must suppress the lived body through harsh trials and leave bodily signals unattended. Breathing through masks awaken feelings of suffocation, and one must control one's breath to manage. Persistent thirst and soreness from tight metal masks that cut into the skin exhaust one's physical resources with each shift. Despite the need for recovery, bodily adaptation develops through the hard circumstances. Difficult and stressful work tasks impossible to unwind from cause an inescapable and constant fatigue. One's senses are weakened by protective equipment, leaving only echoes of what was communicated about the patients, hard to interpret. The exhaustion entails feelings of anxiety and discomfort:

The masks were terrible. I had sores behind my ears and on my nose. And it muffled the sound a lot, plus you had a visor on, so you basically stood and shouted in there to communicate with each other. Then you had to think about your breathing, not to breathe too fast, because then you didn't get much air/…/You had a constant headache from the visor, which was tight. It was hot, you were wearing a full body coat, it was heavy.

*Continuing to work by shutting oneself and the outside world out* mean the presence of death and doomsday might be kept at a distance only by letting the thoughts of the pandemics remain in the hospital. Distancing also entail private life by excluding pandemic coverage. Simultaneously the everyday life continues much as before as there is nobody taking over one's household chores. Shutting down one's mind is necessary to disconnect from thoughts about how badly affected the patients are and the plight of their families:

I don't watch the news anymore; I can't stand it. I am not watching the press conferences, can't stand it.

*Being enclosed in an emotional vacuum* means being so absorbed in the world of the pandemic, leaving emotions too detached to reflect on. The difficult, high‐paced work situation entails no space to reflect in the moment and putting thoughts into words is difficult. Society's disregard to follow restrictions causes frustration and leads to doubts whether the world will ever return to normal.

Details in everyday life, which previously felt insignificant, take on great importance such as being in nature and in the garden which bestow recovery and strength to face another workday. Conversely, what was previously appreciated now must be put aside, i.e., social relationships. In the emotional vacuum sleep must be prioritised and everyday chores are given secondary importance:

You take everything in the moment. Today is today, and tomorrow is tomorrow, and I'm doing my best. One must not think too much. Because then you go crazy.

#### 4.2.3 Feeling Vulnerable and Unsupported

Feeling vulnerable and unsupported includes being forced into a frightening, stressful and demanding reality and being responsible for advanced care without training and feeling left out and enduring challenges alone.

*Being forced into a frightening, stressful and demanding reality* mean the transformed caring reality instantly put former, safe work environment out of reach. To suddenly care for patients with advanced needs is overwhelmingly difficult. Strict requirements alongside with a novel demanding framework subject oneself to struggle and frustration of not getting the necessary support performing the difficult tasks assigned in acute situations. Own skills and resources are not enough for the new, alien work evoking a sense of war. Help is out of reach and there is a desire to escape:

Normally you read about the war in the newspapers… but the war is somewhere else/…/and even if that is close too, now it definitely felt like you were entering the war and then the war was 600 meters away from home. It was emotional…. I did those comparisons, absolutely.

*Being responsible for advanced care without training* means one does not receive proper introduction, must toil hard in unsustainable working conditions, and manage constantly changing directives. Advanced, complex caring situations with no given answers mean demands to go outside the comfort zone and take risks with people's lives at stake. This makes one feel lonely and in loss of control. Doubts as to whether one's own efforts are correct relate to nobody seeming to have time to listen. One lacks understanding of the prerequisites needed to work in this new caring reality and the impact of not having adequate resources and skills:

The thing that was most unknown was the tracheostomies, such an important thing connected to breathing. If it does not work, then it's really urgent. Initially, I didn't know how to take care of it/…/It was only to keep trying to get through it the best way possible, to improvise with whatever tools you had.

Amid all the challenges, the high degree of individual responsibility means a constructive learning process too:

Then I also noticed that I was probably capable of a lot more than I actually knew I was. Now, you have a lot of knowledge that you may not always use on a daily basis. And you have learned an awful lot too. It is an enormous amount of knowledge you have gained.

*Feeling left out and enduring challenges alone* mean having to trust in one's own inner peace and confidence that it would work out despite all the uncertainty. Experiencing misunderstanding and insufficient support mean feelings of confinement and isolation:

I felt completely left out and like it was all fake, like I was pretending, it was so unreal.

One's thoughts and strength to endure abide with feelings of fatigue and exhaustion. Hidden strengths preserving bodily resources can be found by walking in the nature, yoga and meditation. Recovery might also be gained by getting ill from the virus oneself and thus being away from work. Eventually one realises that the current situation cannot not last forever, an important conclusion to maintain hope.

Reflection gives the opportunity to consider one's contribution as significant and accommodating colleagues' reactions mean self‐recognition of one's efforts. Acknowledging colleagues' resources and strengths and recognising the significant impact of the pandemic on them, evokes mixed emotions towards those who chose not to partake in the care for COVID‐19 patients:

I don't think I realized how hard it was with COVID then. That's probably my biggest insight. I'm used to seeing these colleagues/…/They have been so professional, and everything… And then when I saw them now, then it was … Well, it is hard to explain really. It was like they were completely broken down/…/And I probably didn't understand that before I got there.

Within the hospital togetherness and a sense of shared responsibility is present, even if it means a risk to own health or of their loved ones. This also mean new insights into who are one's friends and accountable colleagues. Ambiguous feelings towards colleagues are characterised by understanding on one hand and by questioning on the other. As time passes the new caring reality become more secure and hidden strengths are found. Still, unhealed wounds remain from frightening insights of irreversible human destinies who met death insufficiently cared for. Altogether, the given rewards for one's efforts are unreasonable. Applauds are not enough.

### 4.3 Comprehensive Understanding

In this section the comprehensive understanding gained from the naïve reading and structural analysis is recontextualised in the light of Eriksson's caritative theory to provide a deeper understanding of the results.

The overarching theme of nurses' lived experiences of stress during the pandemic is encapsulated in the metaphor ‘Enduring the unbearable’. Encompassing nurses' prominent altruistic stance to respond to patients' needs despite hard circumstances. Eriksson's caritative theory emphasises the importance of a holistic approach to caring, which includes acknowledging patients' suffering and understanding their needs encompassing the physical, emotional and existential dimensions of the patient's experience. However, inner beliefs and values are often compromised due to the fast‐paced influx of patients, lack of knowledge and high workload during the COVID‐19 pandemic. Despite facing exhaustion amidst the irreversible and frightening transformation in healthcare, nurses silently persevere in their efforts to save lives. The barriers of protective equipment and time constraints hinder them to provide compassionate holistic care, even when the patient is dying. Eriksson [1] describes the starting point of nurses' professionalism as taking wholehearted responsibility for the dignified care of the suffering patient and doing good, emphasising the alleviating of suffering through caring actions, despite resource limitations.

Interpretation of the findings encompass inner and outer horizons of nurses' experiences of stress during the pandemic. Themes highlight how nurses stoically remain in ethically compromising situations, often with their voices silent. On a pragmatic level, multiple stressors form an outer horizon of nurses' lived experiences of stress, severely impacting their physical well‐being. Delving deeper into the analysis, an inner horizon of nurses' lived experiences of stress emerges, revealing the meaning behind having to violate one's core caring values strongly connected to one's moral compass of what is good and bad, right and wrong. Nurses experiences involve ethical struggles and moral distress when they are unable to provide the level of care, they believe is necessary significantly impacting their well‐being and professional identity. Acting against one's moral compass raise questions about professional identity and the consequences of living with lingering memories of stress with severe impact on the lived body. Eriksson's theory suggests that refraining from one's inner beliefs and values can be understood as a loss of freedom. This holistic understanding aligns with the findings of nurses' experiences of encountering suffering and death, and their ethical struggles in providing care under challenging circumstances.

## 5 Discussion

This study aimed to explore nurses' lived experiences of stress in the transformed caring reality during the COVID‐19 pandemic. Being subjected to unfamiliar work tasks, working in harsh protective equipment, putting up with constantly changing, unclear directives and enduring long working hours summarise an outer horizon of stress known from several systematic reviews and meta‐studies [8, 24, 25]. In the light of Eriksson's caritative caring theory [1] our findings support the inner horizon of nurses' stress by nurses' immense sacrifices having to violate the core of caring. Being subjected to questioning the self‐image as a caring, present and compassionate nurse aiming to relieve suffering seems to be utterly stressful. Thus, a lack of goal fulfilment seems to be stressful, rather than a fast‐paced working environment. Our findings support the pivotal importance of nurses' work being experienced as meaningful. Nurses seem to endure immense difficult work circumstances as long it feels meaningful. Meaningfulness connects to holistic ontology and apply to nurses as holistic carers. Our study shows that during the pandemic focus was drawn away from inner core caring values with heavy negative impact on nurses' health and well‐being.

Partaking in unworthy care means questioning one's self‐image as a nurse guided by inner core caring values [1]. This enhances the importance of circumstances enabling nurses' perseverance to care for patients according to caring values even in times of organisational change. Reinforcing the importance of a holistic approach to caring, which includes acknowledging patients' suffering and understanding their needs, connects to nurses' experiences of feeling deprived of their caring self and the impact on their professional identity. Strong quotes reveal how the transformation in healthcare disclosed caring actions in the strive to save lives. When exploring the meaning of caring within emergency care, advocacy and holistic care are expressed as pivotal, thus jeopardized by, that is, lack of management support, chaotic workload, staff shortage and lack of self‐care. Enduring stress is closely connected to the importance of experiencing meaningfulness, manageability and comprehensiveness in their role. However, caring is a natural way of being in the world, inevitable and nothing one can set aside or avoid. Ethical and moral dimensions of caring acknowledge the patients' suffering and departs from Aristoteles' view to altruistically concern for another human being. This can be understood as a dignified act of love from one person to another requiring a holistic approach to human needs and vigilance on how the patient wants to be cared for [1, 2, 3, 4].

Exploring the role of self‐compassion and resilience in coping with stress, Eriksson's theory can support nurses in maintaining their self‐efficacy and job satisfaction despite the challenges they face by finding ‘the why’ in suffering. Less self‐compassion depleted nurses' bodily resources with little possibility of recovery, also verified in other studies [26, 27, 28]. Thereto, partaking in unethical situations created feelings of guilt and self‐blame rather than self‐kindness a dimension within self‐compassion scale [29]. Our findings support the idea that nurses risk their emotional energy being torn between the strive for bestowing compassionate care according to core values and dissonance to its importance in the working environment. Thus, our findings illuminate that to do good for others also is a way to find meaning for the carer. However, long‐term impact on nurses' health by the pandemic is hard to estimate and needs to be further investigated.

Alongside with meaningfulness and self‐compassion, our finding illuminate how nurses try to endure stress by various means, that is, distancing, spending time in nature and exercising. It has been suggested that emotional distancing as a form of self‐protection can have negative implications for the quality of care delivered and the wellbeing of the caregiver by elevating levels of emotional exhaustion and general fatigue, core components of nurses' mental health and healthy work life [30]. However, this shall not be interpreted as pervasively negative, emotional demands can also act as challenges to nurses and promote motivation and well‐being [31]. This aligns to previous research [32] suggesting dimensions of victory and tragedy within nursing care.

In line with Eriksson's theory [1] nurses during the pandemic emphasise holistic care, ethical decision‐making and the importance of meaningfulness in their work. The presence of a worldwide pandemic does not alter the essence of quality care. Current trends in healthcare focusing on patient‐centred care, resilience and the well‐being of healthcare professionals must be addressed sustainably to maintain effectiveness in crisis situations. Benner and Wrubel [2, 32] highlight the concept of ontological caring to endure stress in caring, which is also supported by contemporary research [10]. This confirms that nurses' caring role are central to improve staffing especially within emergency care [33]. Emphasising the need for organisational support, such as constructive leadership and person‐centred care, helps nurses cope with stress. The complex phenomenon of caring has been thoroughly investigated and valued from the patients' perspective; yet it is still underestimated as a health promoter for nurses. According to this study, it is crucial to acknowledge nurses in their role as caring professionals when aiming to understand how to support their endurance in a stressful work environment. Caring research frequently explores the outer horizon, a conceptual description of stress. However, our findings indicate that the inner horizon of stress is shaped by the inability to care according to one's inner ethical compass. We argue that both inner and outer dimensions of the phenomenon of stress need to be embraced in research and in practice. Future research should further investigate the ethical dimensions of stress and its impact on nurses.

### 5.1 Strengths and Limitations

Aiming to reveal the interpreted meaning of nurses' lived experiences all researchers have been inspired by a hermeneutical lifeworld approach. Guided by phenomenological hermeneutical method as described by Lindseth and Norberg [22, 23] the lifeworld perspective was chosen to mirror the lack of research focusing on lived experiences among nurses in Swedish research during the pandemic. According to hermeneutical philosophy issues of stringency to rigour is important. Embracing a lifeworld perspective in caring science entails to without diminishing, belittling or adjustments explore, interpret and describe the subjectively experienced and qualitative world as it is lived [34] undertaken by maintaining an open and compliant approach. By quotations this study carries unique meanings from the participants. All researchers are well familiar with interpretative research and two of the researchers worked together with preliminary analyses in close collaboration between all researchers. Thereto, despite following the methodological steps to create distance to authors' pre‐understanding of experiences in emergency healthcare, this cannot be completely ignored, but there were also researchers with other backgrounds, which means an ability to discuss from different perspectives to open up a new understanding [23].

Important to be emphasised in the discussion about the credibility of qualitative research results, all interpretations of reality are subjective and written texts always contains several possibilities for interpretation. However, according to hermeneutical philosophy issues stringency to rigour is more important than traditional discussions of validity and reliability. Awareness of new comprehensive understanding coming from the most reasonable interpretations rather than arguing ‘the truth’ [23]. By reflecting on the understanding when different levels of interpretation are set against each other in the research group and relating to the literature a new comprehensive understanding emerged. Being conducted in an emergency care context in one hard hit region, transferability of these findings need to be undertaken with precaution into other settings.

## 6 Conclusions

Nurses' lived experiences of stress during the COVID‐19 pandemic accommodate the two‐folded dilemma of having to endure immense work life stressors and stress from not having the prerequisites to bestow holistic care. On one hand, this dilemma contains an outer horizon including multiple stressors such as harsh protective equipment, constantly changing directives and long working hours. On the other hand, the inner horizon encompasses ethical struggle and moral distress when nurses face obstacles to provide the level of care, they believe is necessary. Nurses stoically remained in ethically compromising situations with their own voices silent. The moral distress they experiences impacted heavily and persistently on their well‐being and professional identity. Findings highlight the pivotal importance of meaningfulness in nurses' work and suggest that ethical dimensions of stress and its impact on nurses need to be further investigated from a lifeworld perspective.

This study emphasizes the need for organisational support and calls for constructive leadership and person‐centred care to help nurses cope with stress. Self‐compassion plays a crucial role in maintaining nurses' resilience and self‐efficacy to develop strategies and methods to uphold meaningfulness in their roles.

## Author Contributions

All authors recurrently reviewed the manuscript and contributed to its improvement, read and approved the final manuscript.

## Ethics Statement

This research received ethical approval from the Swedish Ethical Review Authority (dnr 2020‐05556) and was conducted in compliance with the Swedish Ethical Review Act (SFS 2003:460) following the principles in the Declaration of Helsinki [35]. The study was approved by the head of the department and each participant reviewed information about the purpose and procedures of the study, including assurance of confidentiality.

## Consent

All participants provided informed consent in writing beforehand.

## Conflicts of Interest

The authors declare no conflicts of interest.

## Data Availability

Datasets are available from the corresponding author upon reasonable request.
